# Supplementary material for: Heterogeneous intercalated metal-organic framework active materials for fast-charging non-aqueous Li-ion capacitors
Source: Nat Commun. 2023 Mar 16;14:1472. doi: 10.1038/s41467-023-37120-9 (PMC10020440; doi:10.1038/s41467-023-37120-9)
Supplement: Supplementary file 1 — Supplementary Information [file 41467_2023_37120_MOESM1_ESM.pdf]

Supplementary Information for

Heterogeneous intercalated metal-organic framework active materials for fast-charging non-aqueous Li-ion capacitors

Nobuhiro Ogihara[1]*, Masaki Hasegawa[2], Hitoshi Kumagai[1], Riho Mikita[3], and Naoyuki Nagasako[1]

[1] Nobuhiro Ogihara Research Group, Frontier Research Management Office, Toyota Central R&D Labs., Inc. Nagakute, Aichi, 480-1192, Japan

[2] Fuel Cell Research-Domain, Emerging Electrification Technology Div., Toyota Central R&D Labs., Inc. Nagakute, Aichi, 480-1192, Japan

[3] Secondary Batteries Research-Domain, Emerging Electrification Technology Div., Toyota Central R&D Labs., Inc. Nagakute, Aichi, 480-1192, Japan

***Corresponding author**

E-mail: ogihara@mosk.tytlabs.co.jp

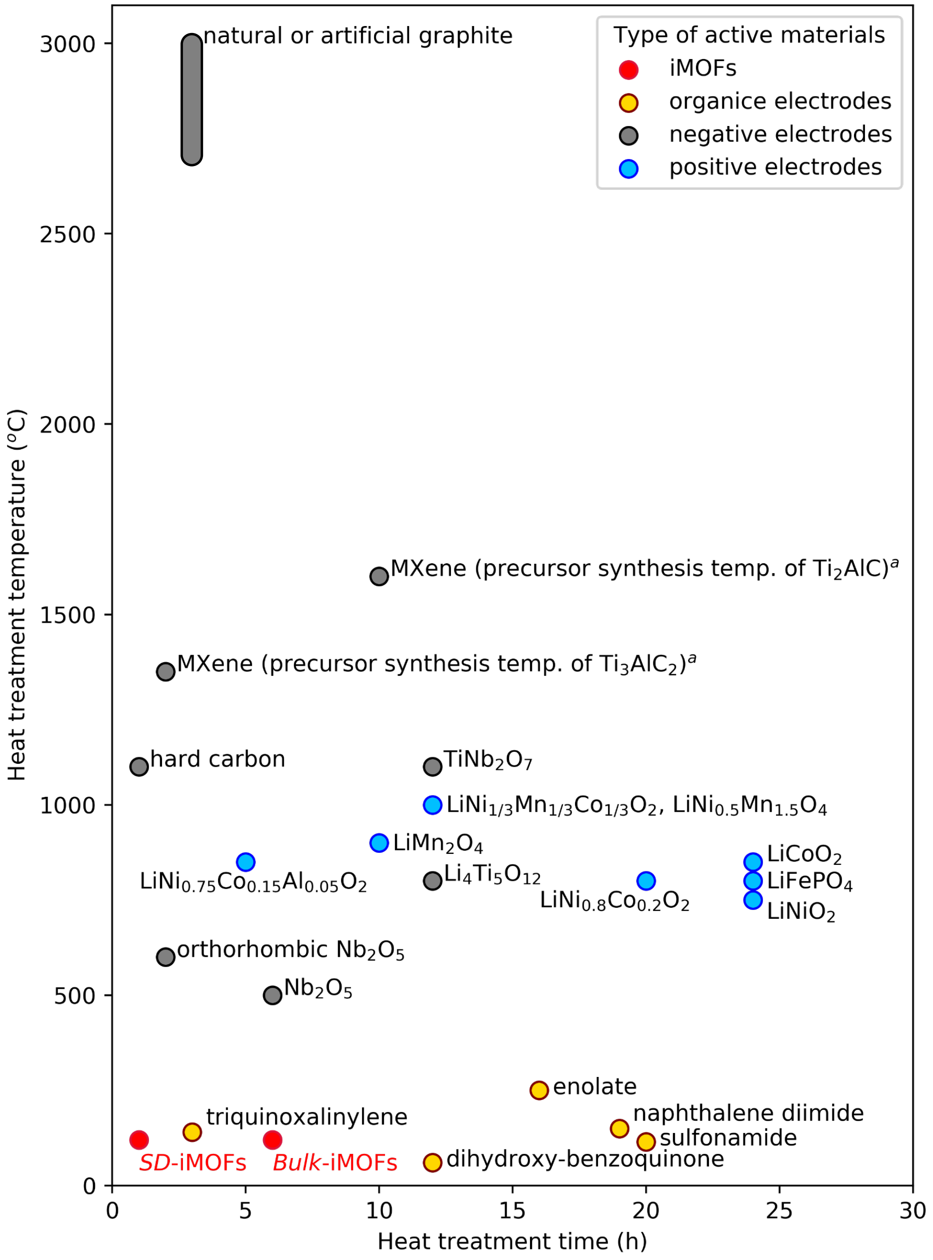

Supplementary Fig. 1. Comparison of heat treatment times and temperatures for electrode materials at the representative specific conditions. Detailed information is given in Supplementary Table 1.

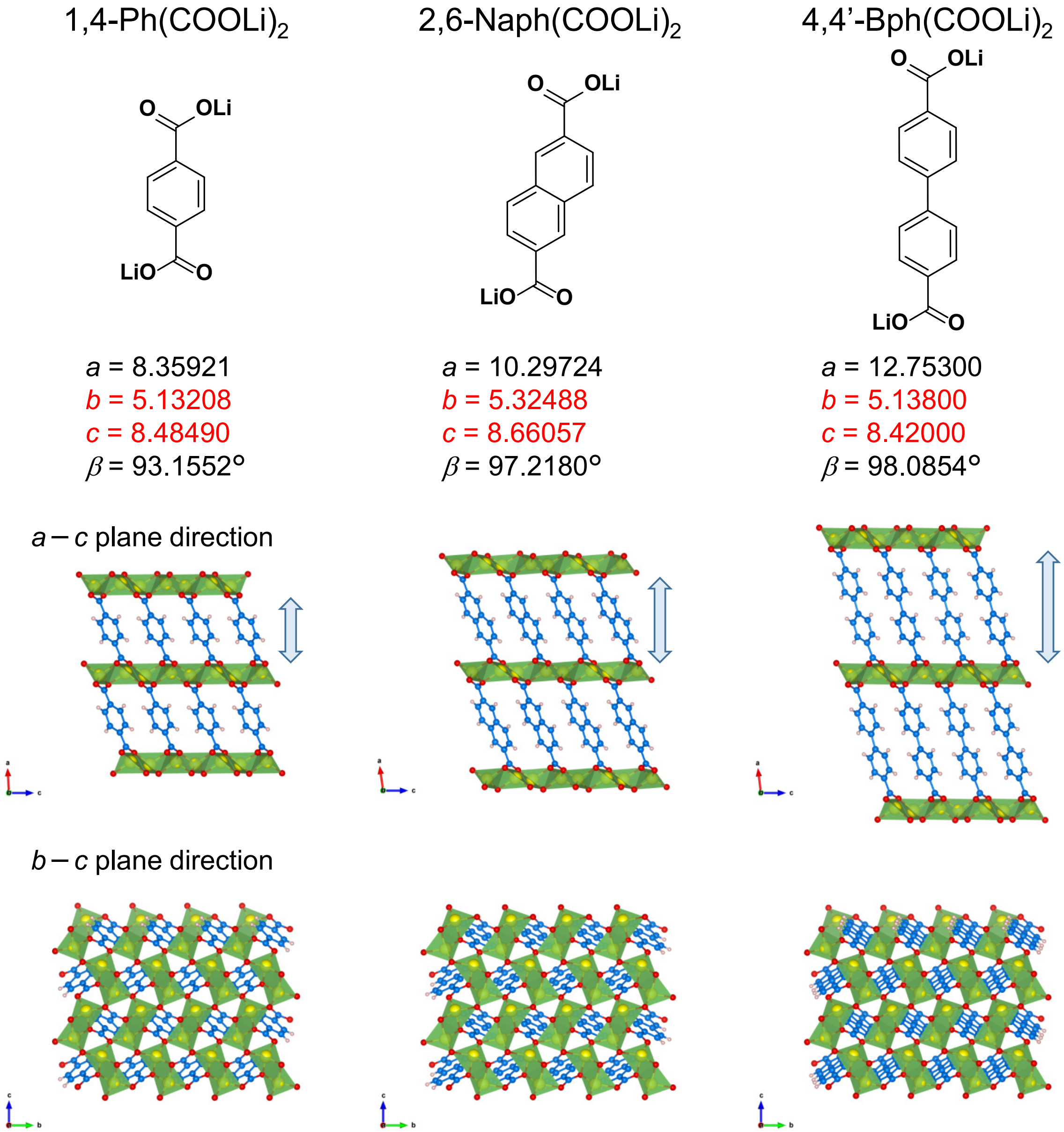

Supplementary Fig. 2. Lattice constants and crystal structures of each aromatic group. Blue, red, pink, and yellow spheres represent C, O, H, and Li, respectively. Green represents the tetrahedral LiO_4 layer.

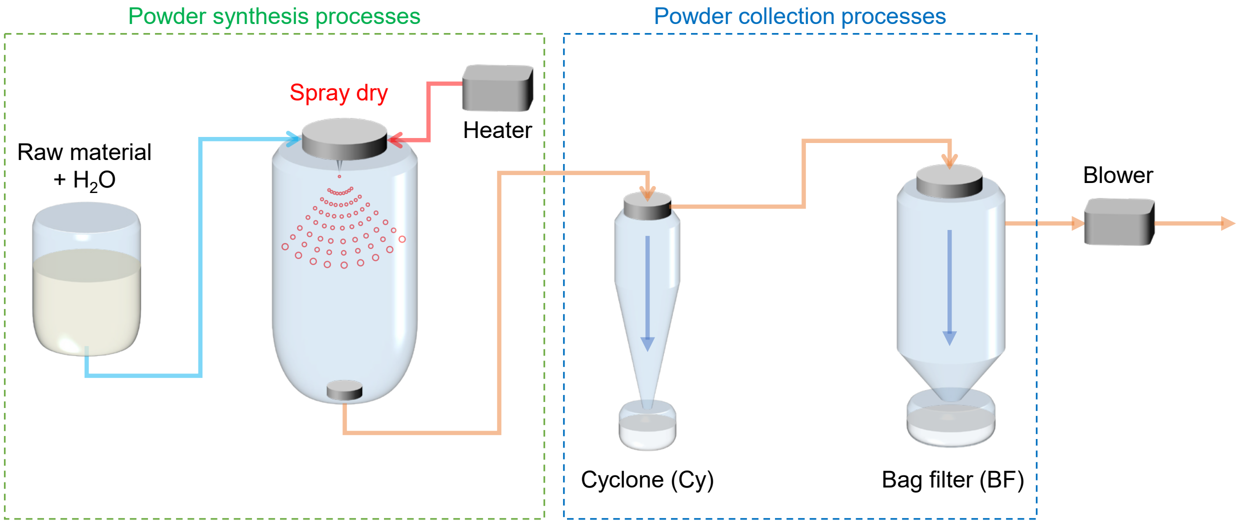

Supplementary Fig. 3. Schematic illustration of spray-drying synthesis. The powders are collected by the cyclone (Cy) and bag filter (BF) equipment.

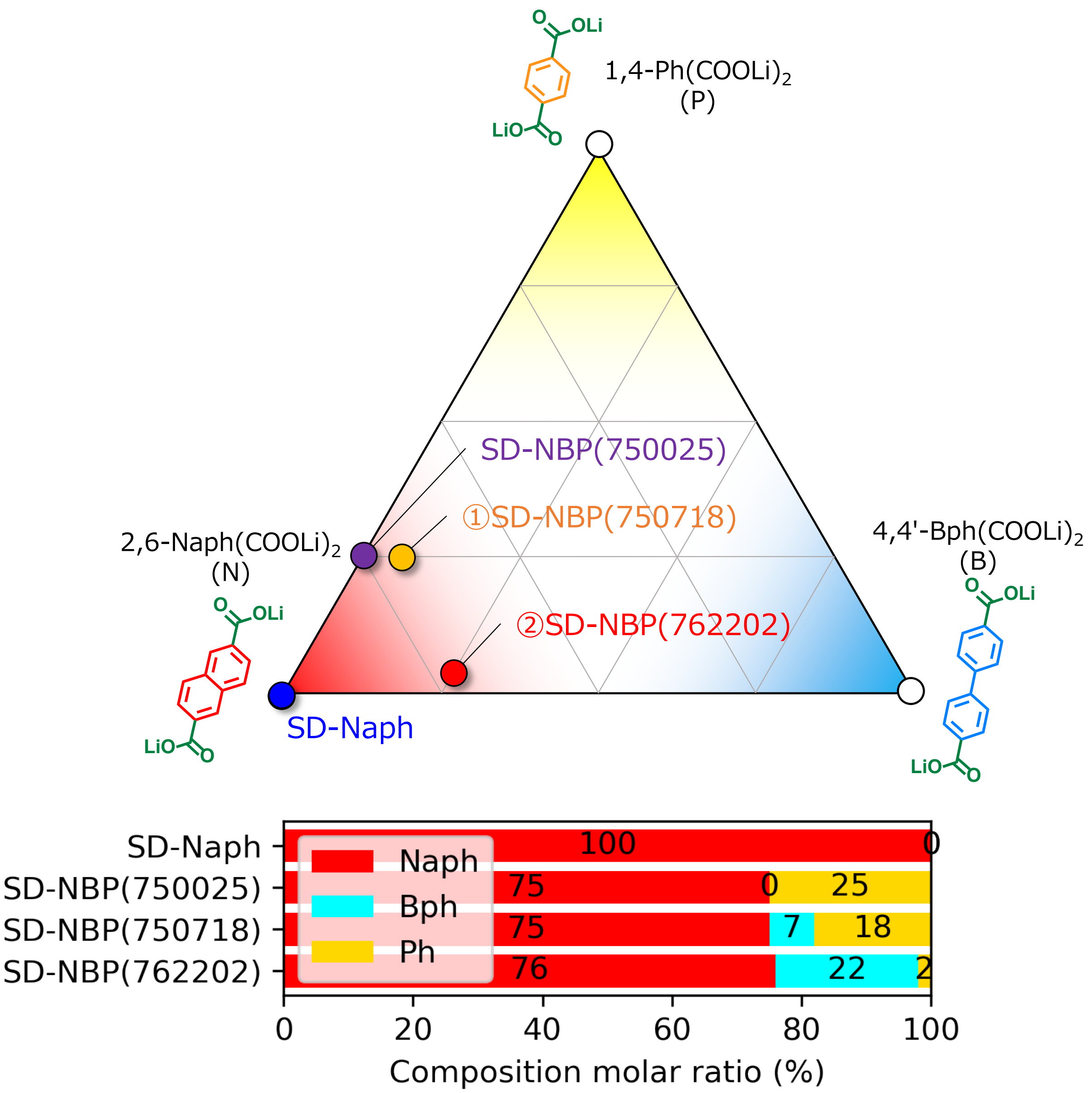

Supplementary Fig. 4. Ternary Ph-Naph-Bph contour map[1] and points and composition molar ratios obtained by machine learning prediction vs. comparative compositions.

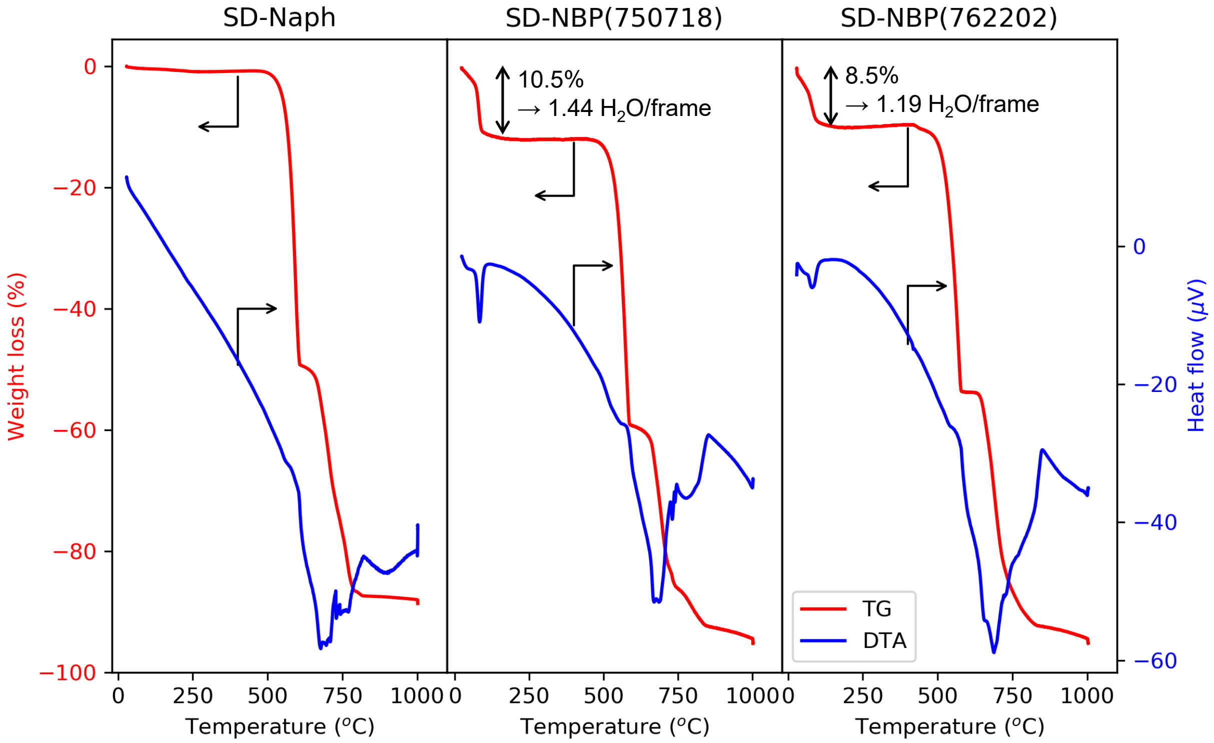

Supplementary Fig. 5. TG-DTA for pristine samples before heat treatment.

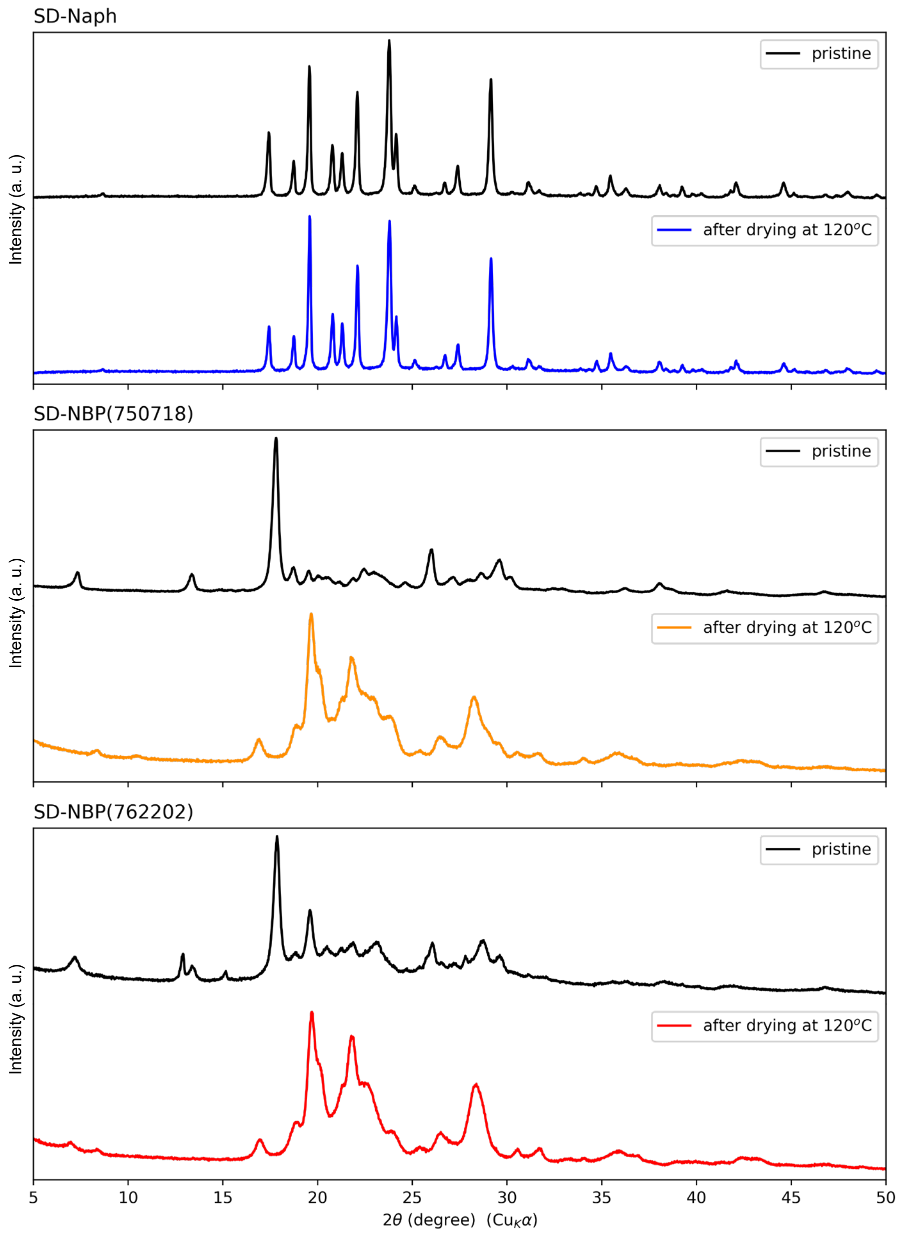

Supplementary Fig. 6. Powder XRD patterns before and after vacuum drying at 120°C.

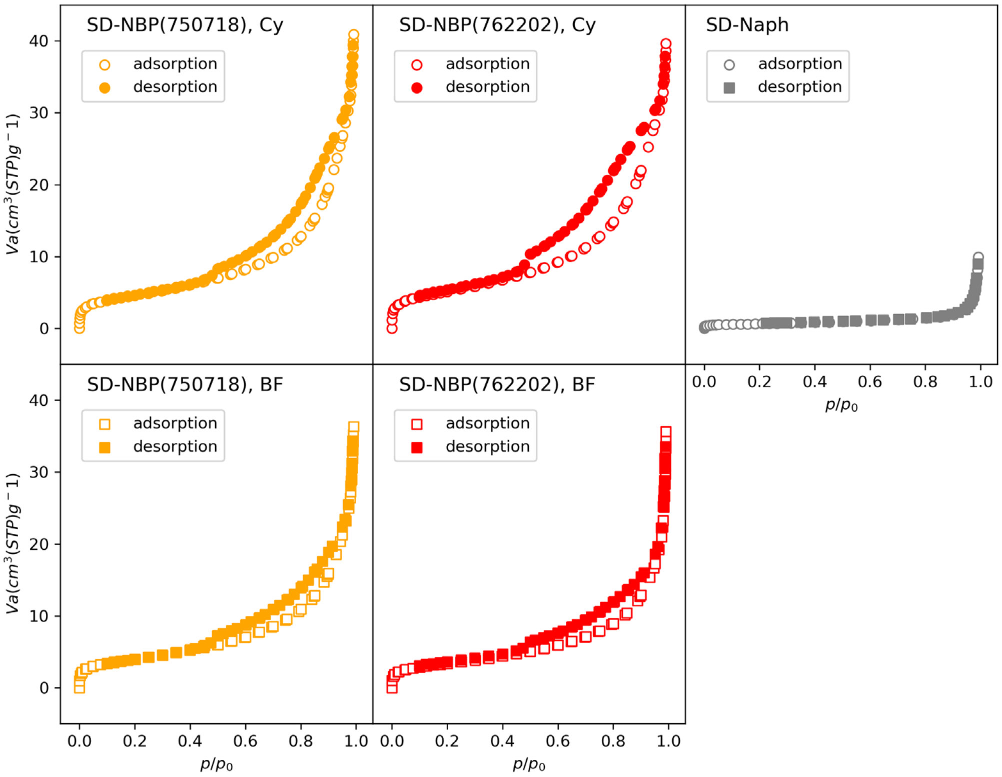

Supplementary Fig. 7. N_2 adsorption and desorption isotherms at 77 K for pristine samples after heat treatment.

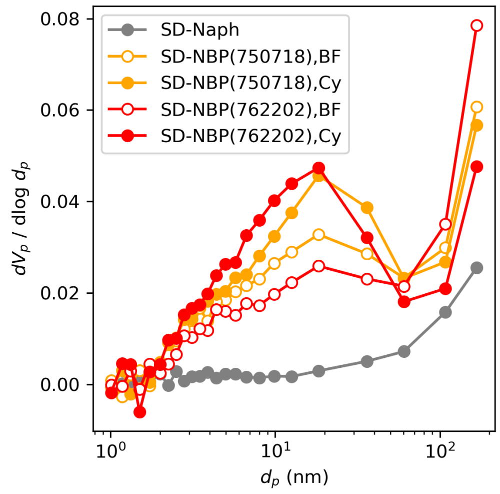

Supplementary Fig. 8. BJH pore size distribution plots.

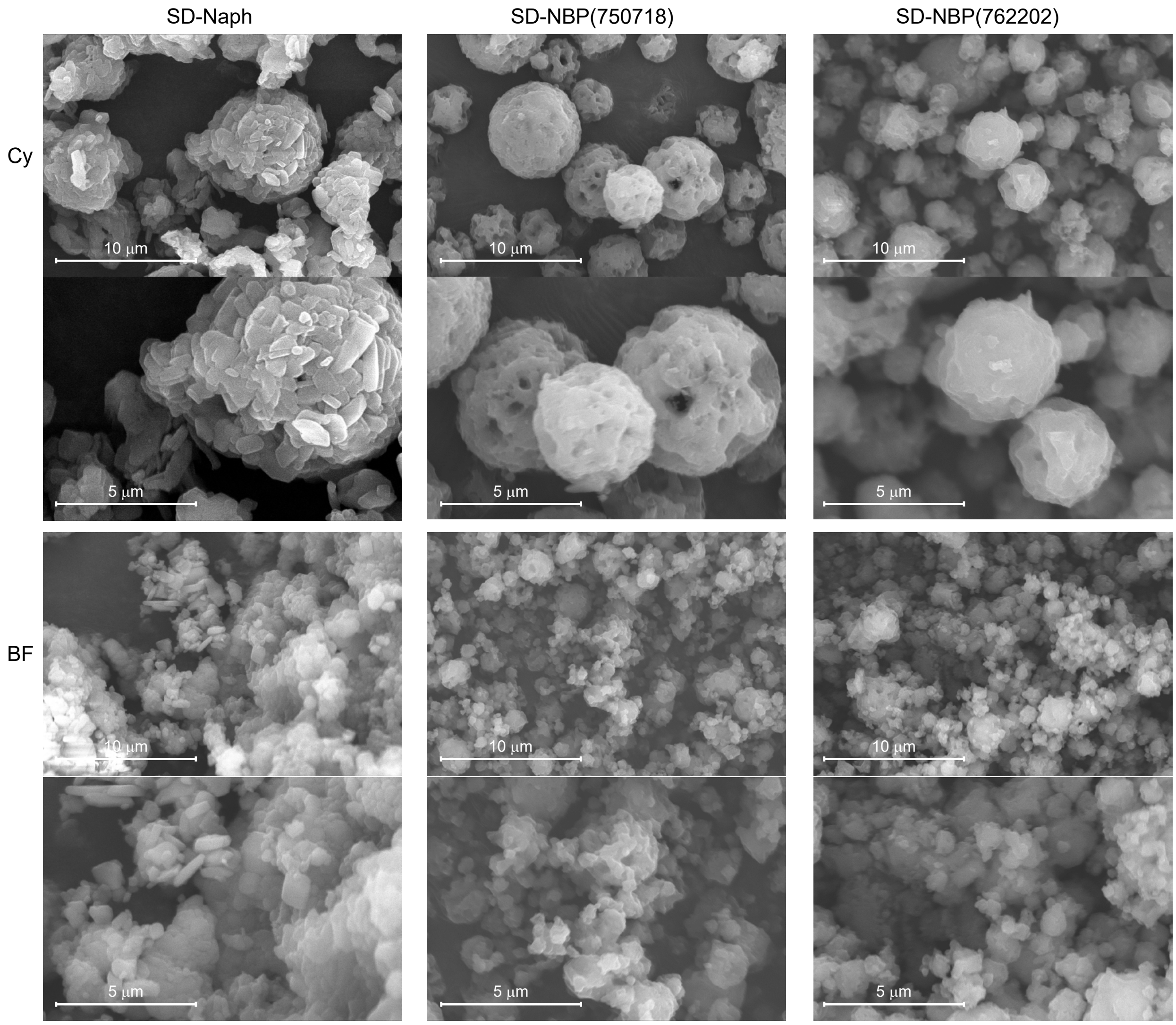

Supplementary Fig. 9. SEM images of pristine sample powders.

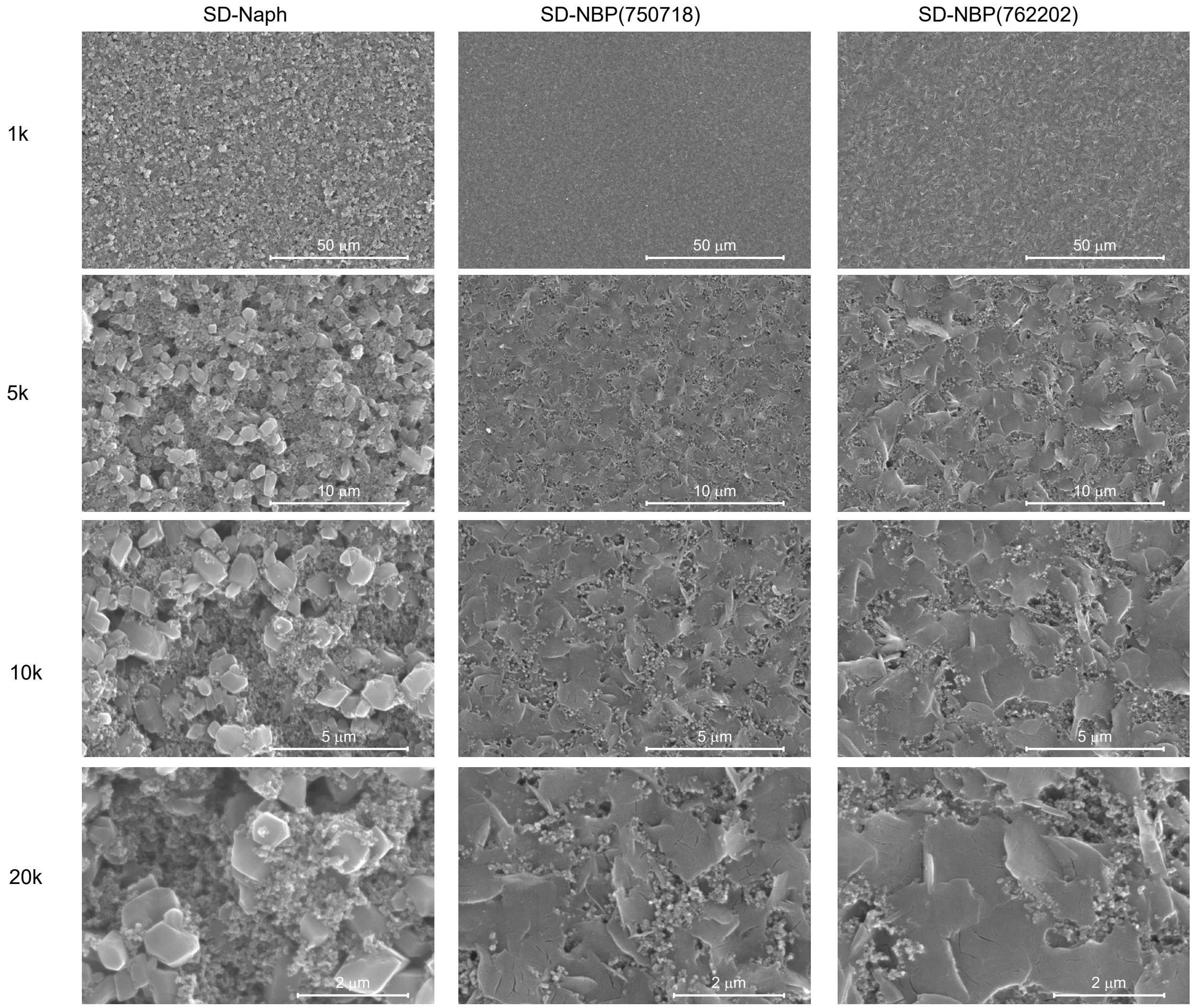

Supplementary Fig. 10. SEM images of pristine sample electrodes.

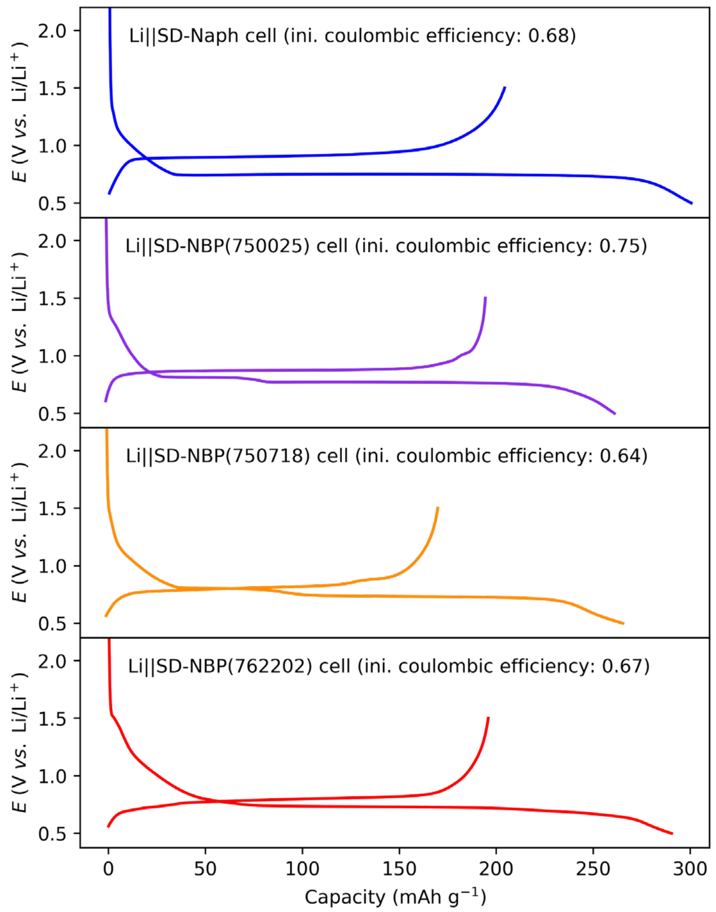

Supplementary Fig. 11. Initial charge and discharge potential profiles in laminate-type pouch Li||iMOF cells using LiFSI-based electrolyte at 20 °C.

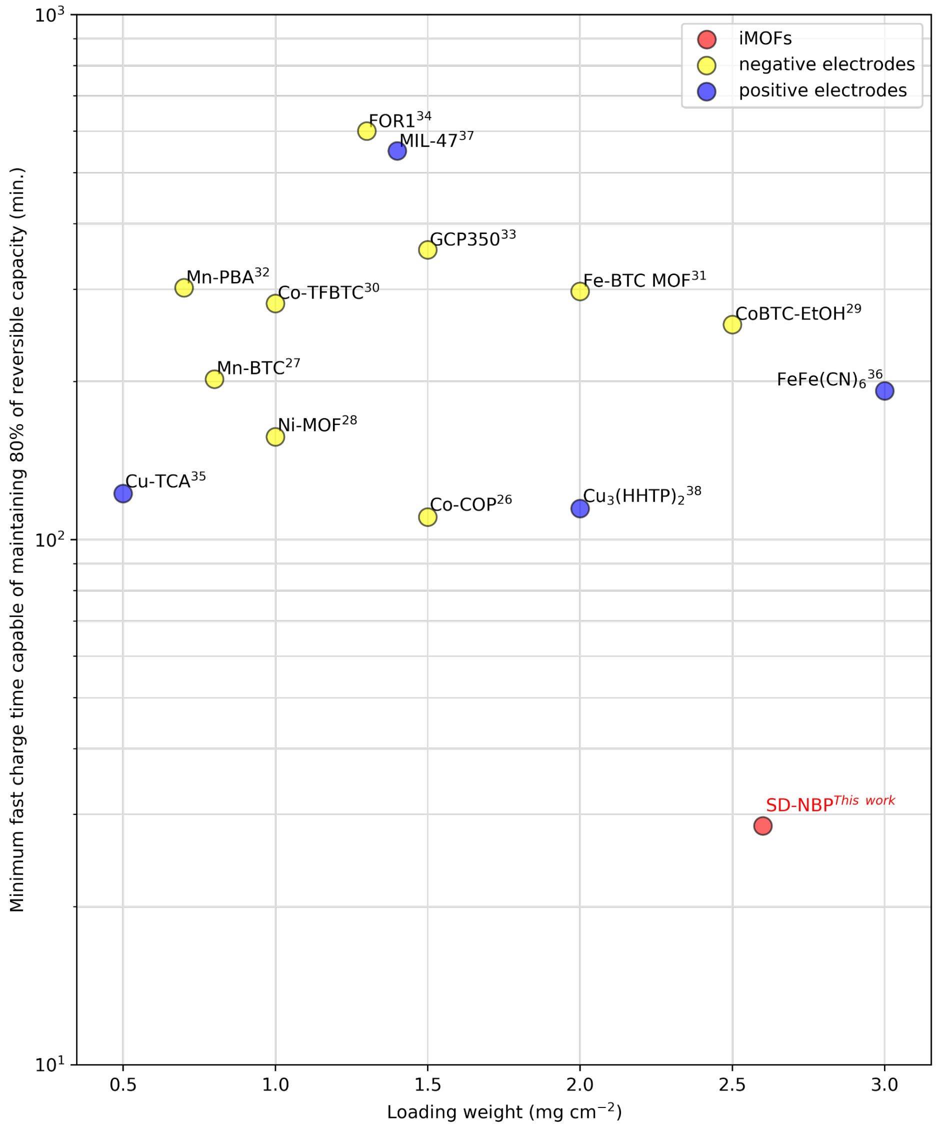

Supplementary Fig. 12. **Comparison of the minimum fast-charging time that can maintain up to 80% of reversible capacity relative to the loading weight.** Detailed information is given in Supplementary Table 4. Fast charging performance for loading weight is important in device design and was compared using the above metrics. Higher loading weight and shorter charging time performance is desired.

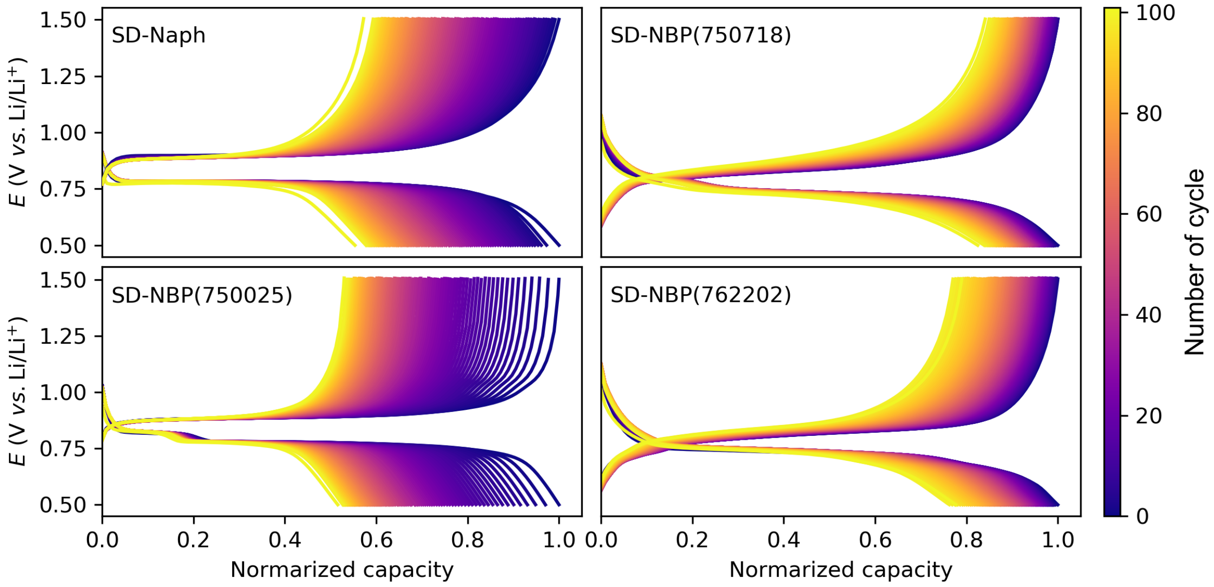

Supplementary Fig. 13. Charge–discharge curves during cycling of the respective laminate-type pouch Li||iMOF cells using the LiFSI-based electrolyte at 20 °C.

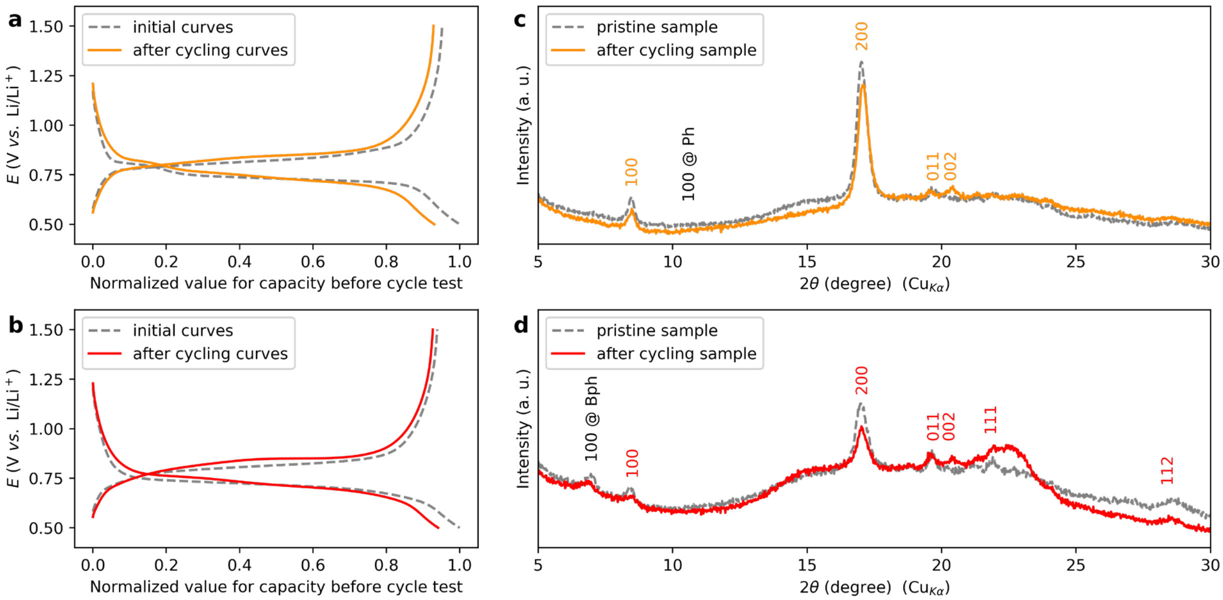

Supplementary Fig. 14. a, b Charge–discharge curves at a specific current of 20 mA g^{-1} at 20 °C for laminate-type pouch Li||iMOF cells using LiFSI-based electrolyte with SD-NBP(750718) (a) and SD-NBP(762202) (b) before and after cycle tests. c, d XRD patterns of SD-NBP(750718) (c) and SD-NBP(762202) (d) electrodes in the Li de-intercalation state before and after cycle test. The peak intensity of the XRD pattern for the electrode state differs from that of the powder in Fig. 1b due to the orientation of the sample[2]. An unidentified broad peak, presumably due to side reaction, was observed around 22° at the SD-NBP(762202) electrode after the cycle test. The peak appearance may be related to the slight difference in cycling performance between SD-NBP (762202) and SD-NBP (750718). Although the XRD pattern after cycling showed a decrease in peak intensity compared to that before cycling, there were no significant changes in the charge-discharge curves at low rates and the peak positions in the XRD patterns before and after cycling, suggesting that such a decrease in peak intensity is due to the influence of the film formed by the reductive decomposition of the electrolyte during charge-discharge.

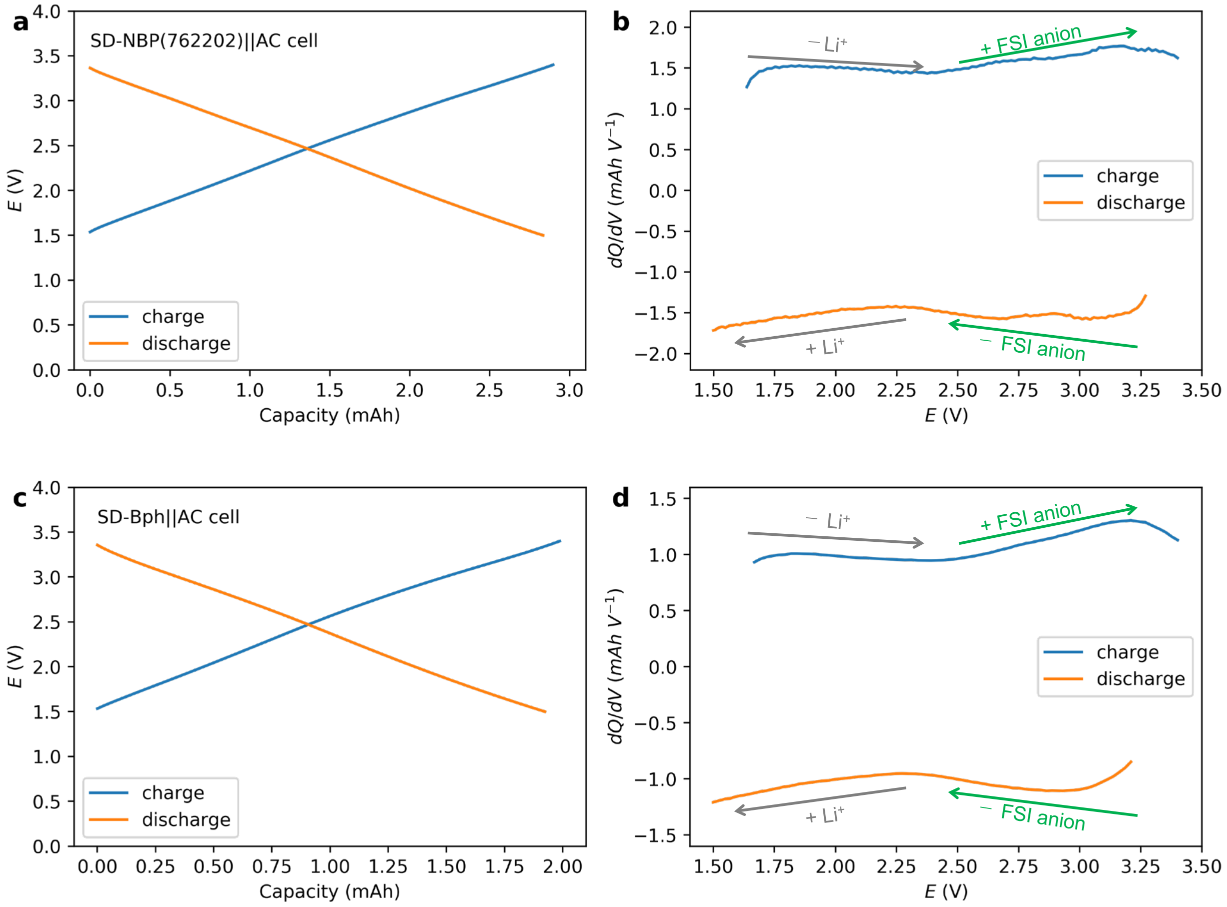

Supplementary Fig. 15. Initial charge-discharge curves (a, c) and their differential capacitance *dQ/dV* plots (b, d) for laminate-type pouch SD-NPB(762202)||AC (a, b) and SD-Bph||AC (c, d) cells using the LiFSI-based electrolyte at 20 °C, respectively. The differential capacitance *dQ/dV* plots (Supplementary Fig. 15b, d) exhibited a typical butterfly-like shape suggesting electric double layer formation of both anions and Li^+ ions[3]. This is evidence that the fabricated cell operates with the targeted reaction mechanism.

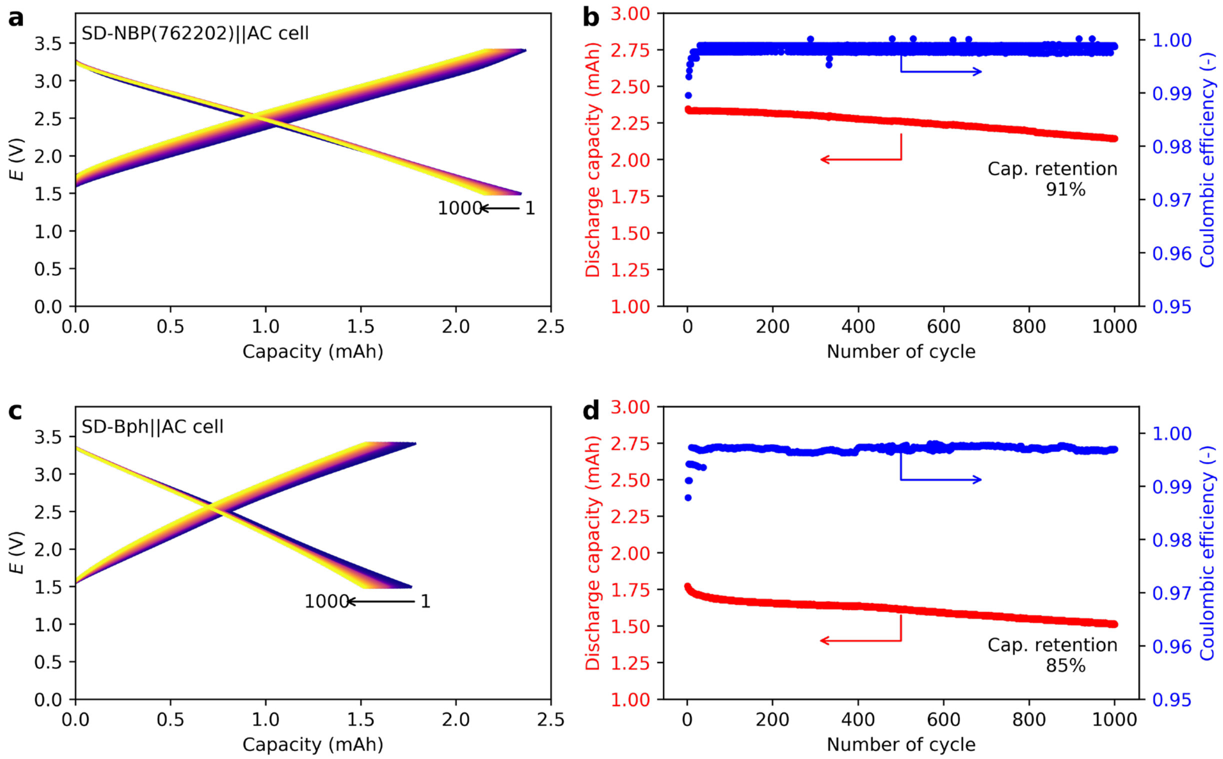

Supplementary Fig. 16. The charge-discharge curve changes at 1000 cycles (a, c) and their discharge capacity changes and charge-discharge coulombic efficiency (b, d) for laminate-type pouch SD-NPB(762202)||AC (a, b) and SD-Bph||AC (c, d) cells using the LiFSI-based electrolyte at 20 °C, respectively.

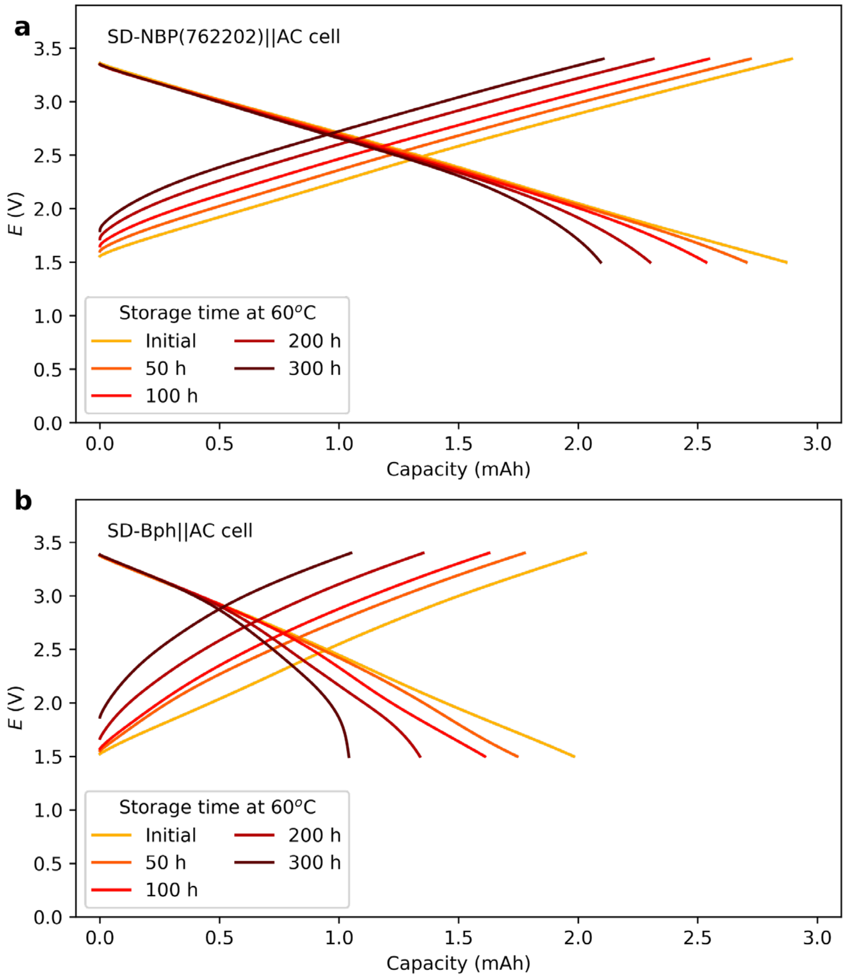

Supplementary Fig. 17. a, b The charge-discharge curve changes at 20°C for laminate-type pouch SD-NPB(762202)||AC (a) and SD-Bph||AC (b) cells using the LiFSI-based electrolyte at 20 °C at various times after storage at 60°C.

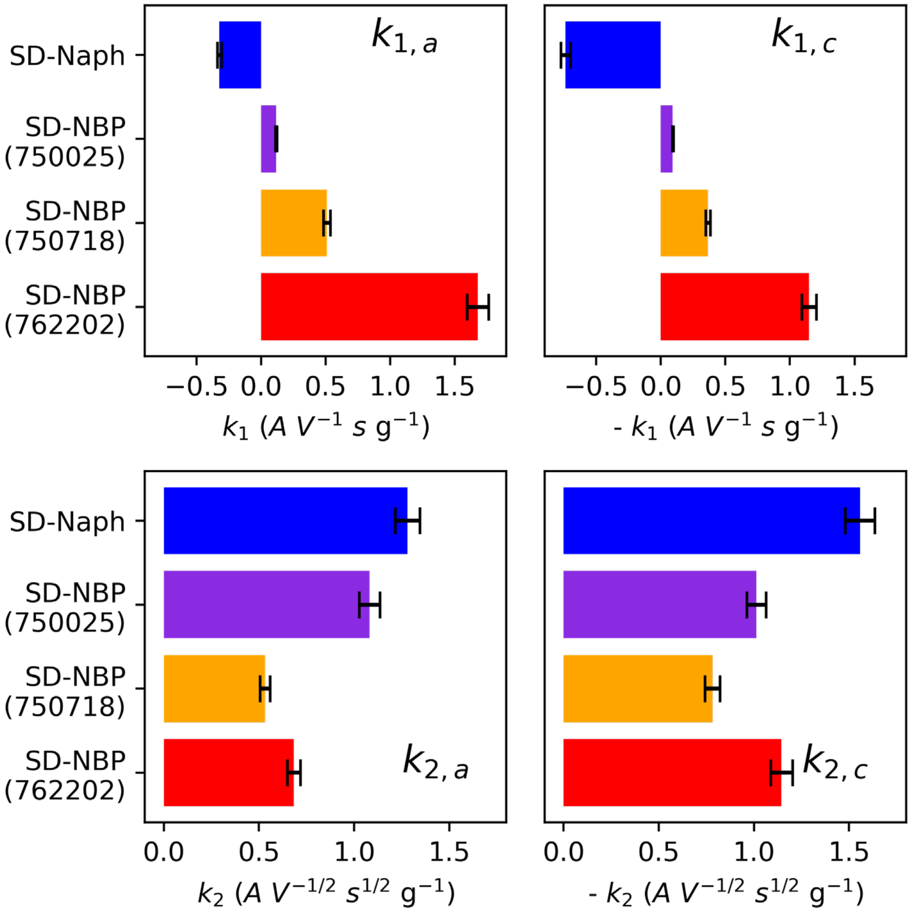

Supplementary Fig. 18. Relationship between k_1 and k_2 in the anodic oxidation ($k_{1,\,a}$ and $k_{2,\,a}$) and cathodic reduction ($k_{1,\,c}$ and $k_{2,\,c}$) directions obtained from the scan rate dependence in CV with Li cells for each sample. Error bars represent standard deviation.

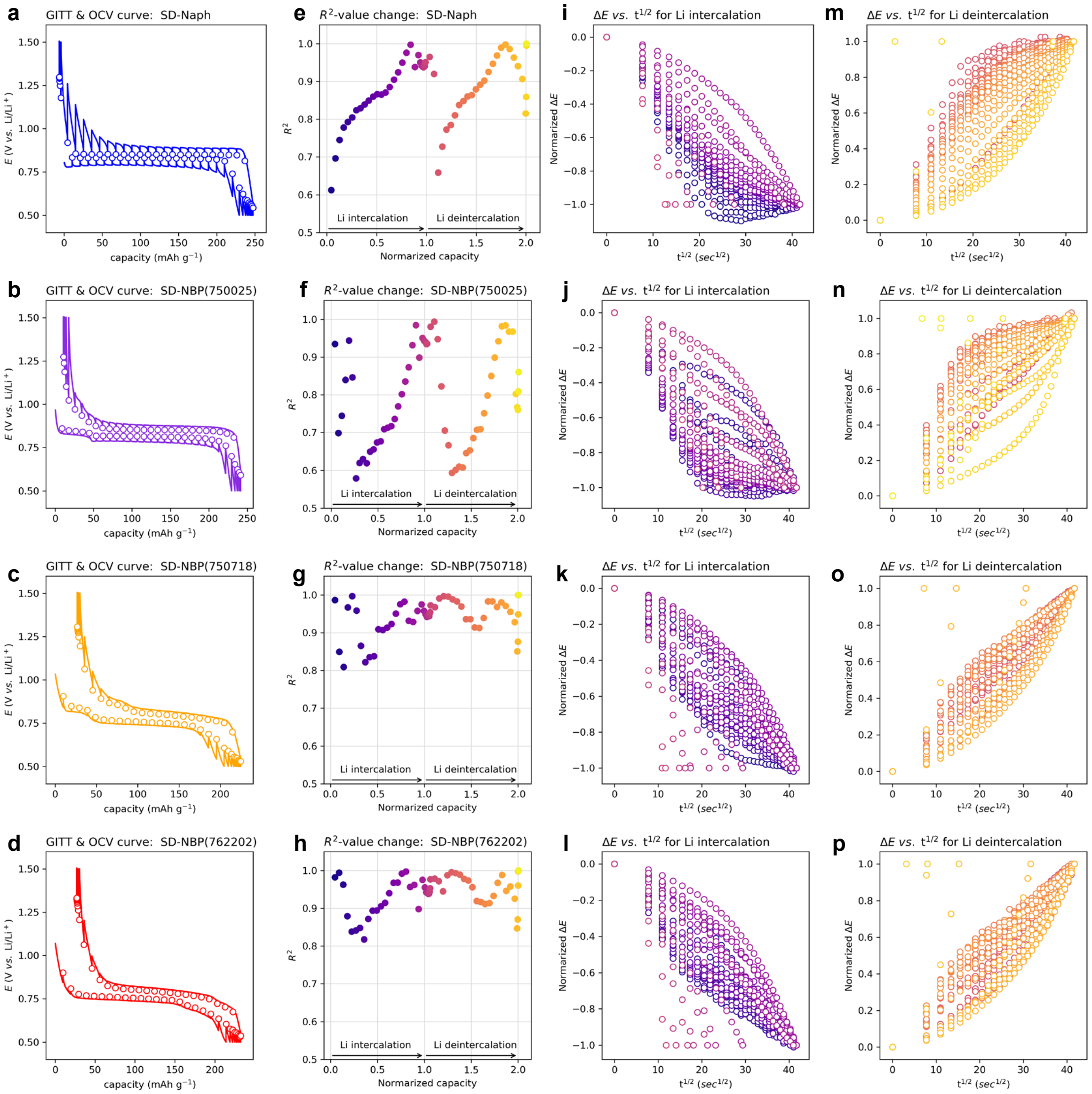

Supplementary Fig. 19. Detailed behaviour of GITT for Li||sample cells. a-d OCV curves for the GITT. **e-h** Coefficient of determination (R^2) in the linear approximation at the root of time ($t^{1/2}$). **i-p** Normalized transient voltage change (ΔE), which includes IR-drop and the potential change upon applying the pulse current (ΔE_τ), at applied current vs. $t^{1/2}$ during Li intercalation (i-l) and Li deintercalation (m-p) for each laminate-type pouch Li||sample cells using the same electrolyte at 20°C.

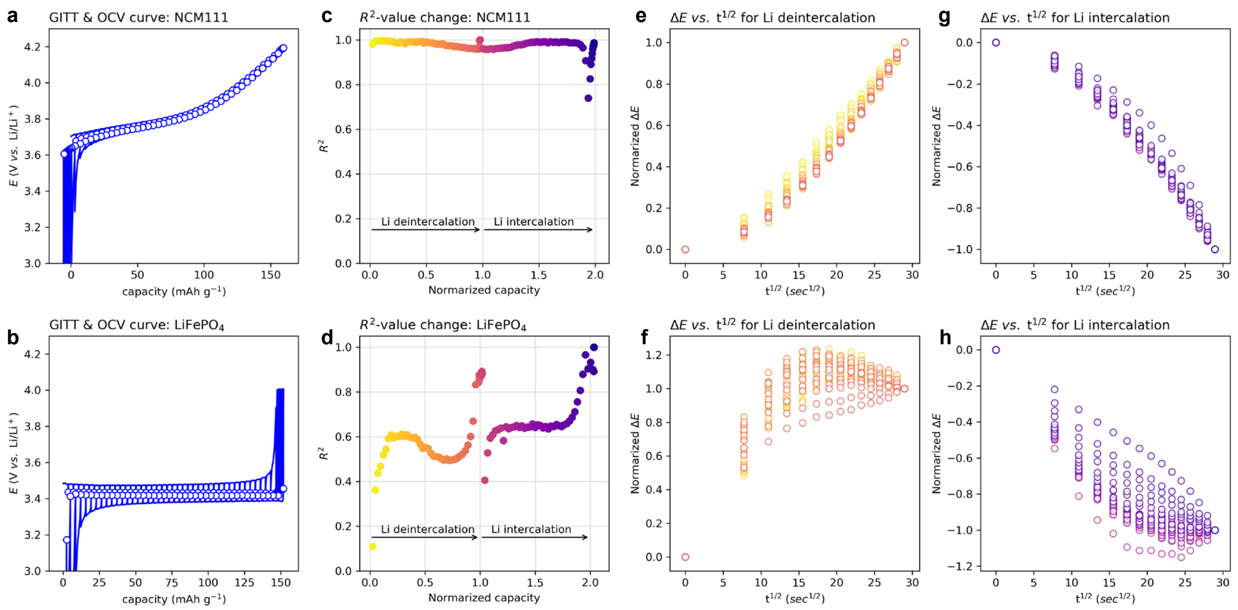

Supplementary Fig. 20. GITT results for Li||$LiNi_{1/3}Co_{1/3}Mn_{1/3}O_2$ (NCM111) and Li||$LiFePO_4$ cells as representative examples of solid solution and two-phase coexistence reactions, respectively. **a**, **b** OCV curves at GITT, **c-h** R^2 at $t^{1/2}$ (c, d) and normalized transient voltage (ΔE) at applied current vs. $t^{1/2}$ during Li deintercalation (e, f) and Li intercalation (g, h) for each laminate-type pouch Li||sample cells using $LiPF_6$-based electrolyte at 20°C.

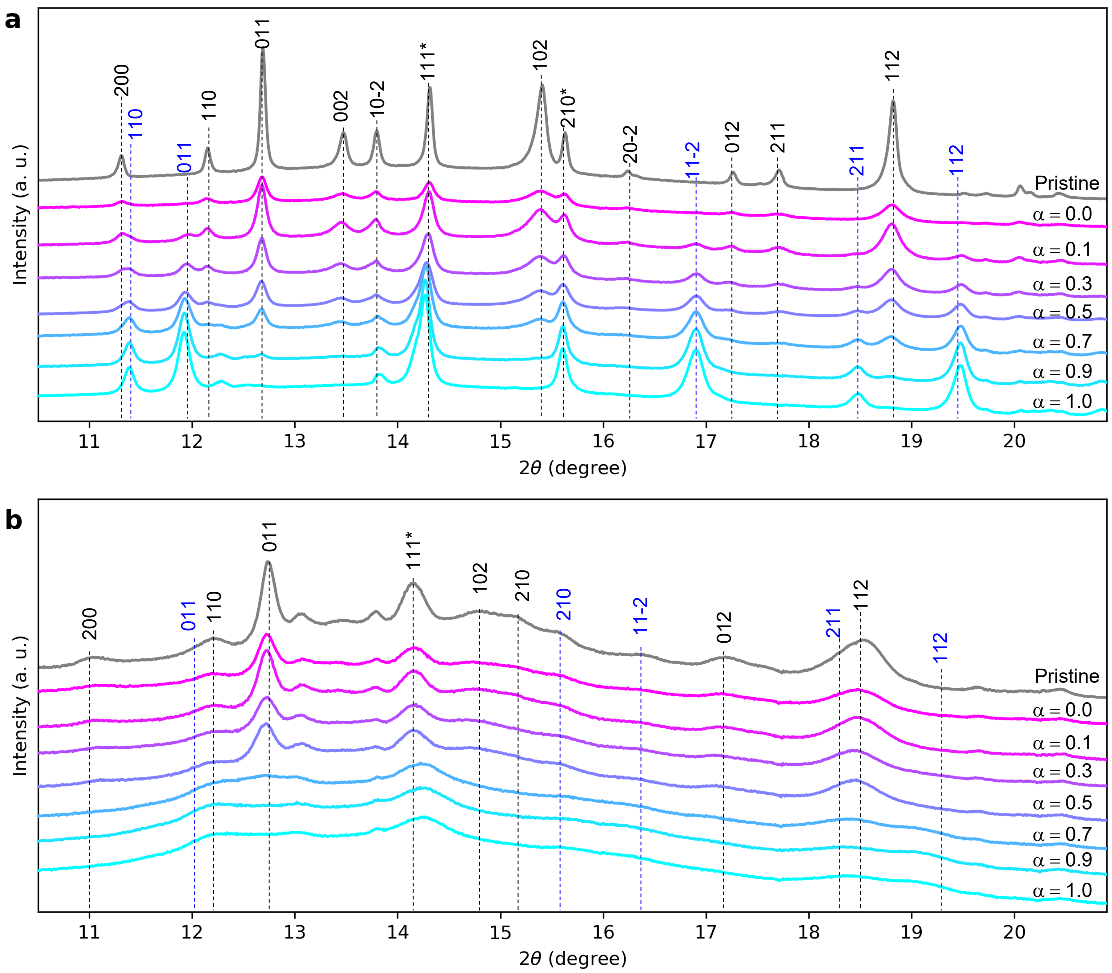

Supplementary Fig. 21. a, b High angle details of ex situ XRD patterns for SD-Naph (a) and SD-NBP(762202) (b) during Li intercalation reaction. The respective samples were prepared using laminate-type pouch Li||sample cells using the LiFSI-based electrolyte at 20 °C. The Li intercalation ratio (α) was defined as the capacity ratio normalized by the reversible capacity.

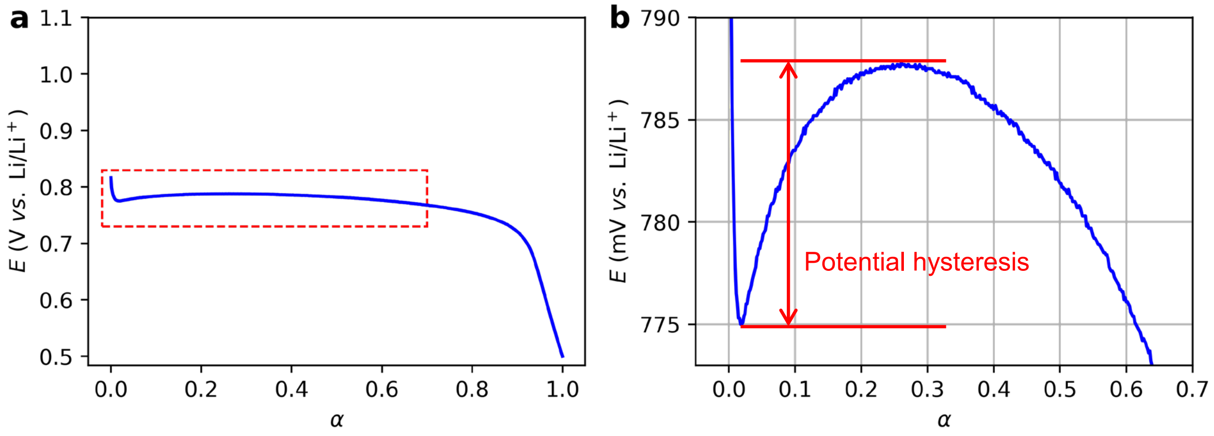

Supplementary Fig. 22. a, b, Discharge potential profile (a) corresponding to Li intercalation in laminate-type pouch Li||SD-Naph cell using the LiFSI-based electrolyte at 20 °C and its magnification (b). The α was defined as the capacity ratio normalized by the reversible capacity.

\

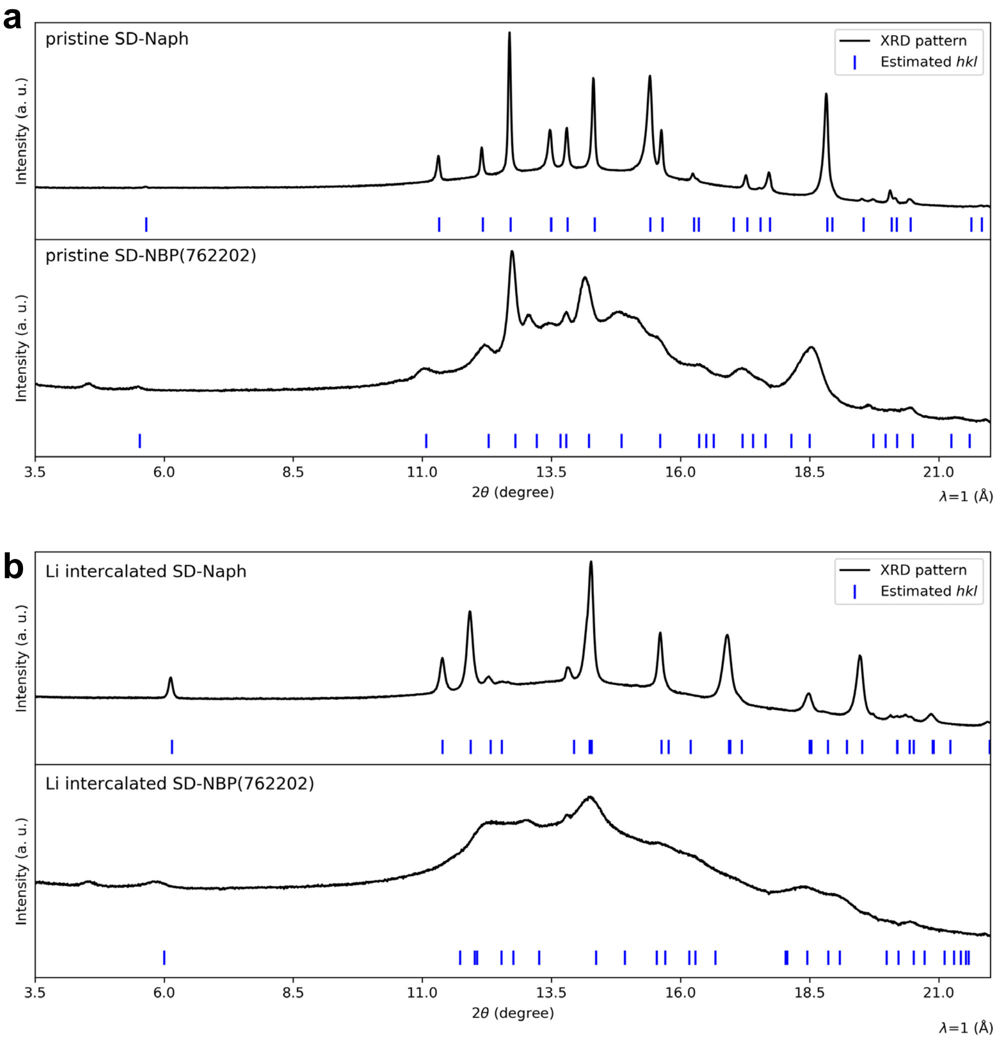

Supplementary Fig. 23. a, b XRD patterns and hkl estimated for a naphthalene framework for SD-Naph and SD-NBP(762202) in pristine (a) and fully Li intercalation states (α = 1.0) (b). The respective samples were prepared using laminate-type pouch Li||sample cells using the same electrolyte at 20 °C.

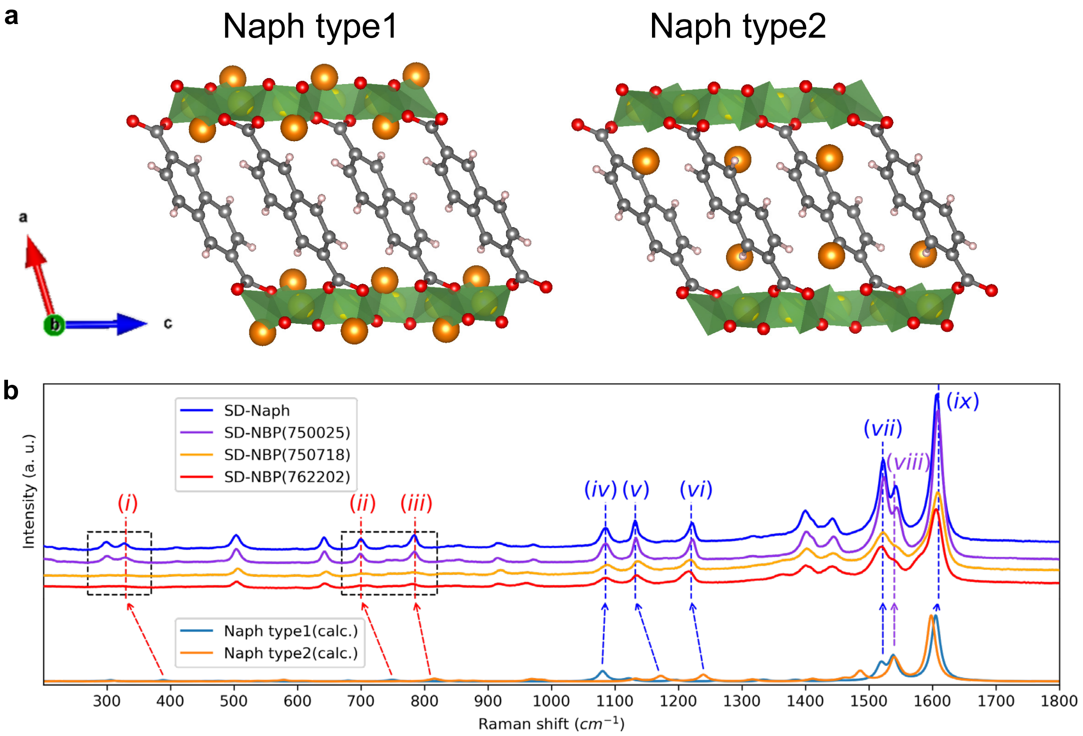

Supplementary Fig. 24. a Crystal structure model of naphthalene-based framework during Li intercalation for phonon calculations. b Calculated and experimental Raman spectra for each fully lithiated state. Gray, red, pink, yellow, and orange spheres represent C, O, H, pristine Li^+, and intercalated Li^+, respectively. Green represents the tetrahedral LiO_4 layer. The crystal structure models during Li intercalation were type 1 and type 2, which were obtained in the previously reported case, and near the LiO_4 tetrahedron and between the naphthalene π stacks, respectively. From the comparison of the peaks of each type 1 and 2 obtained by phonon calculations, the Raman shift was corrected to match the vibrational modes in each type. The difference between the experimental and calculated values can be attributed to the equilibrium volume and lattice volume, which are related to the bond lengths between atoms, and these factors are influenced by the temperature and Li site occupancy, which were not considered in the present calculations.

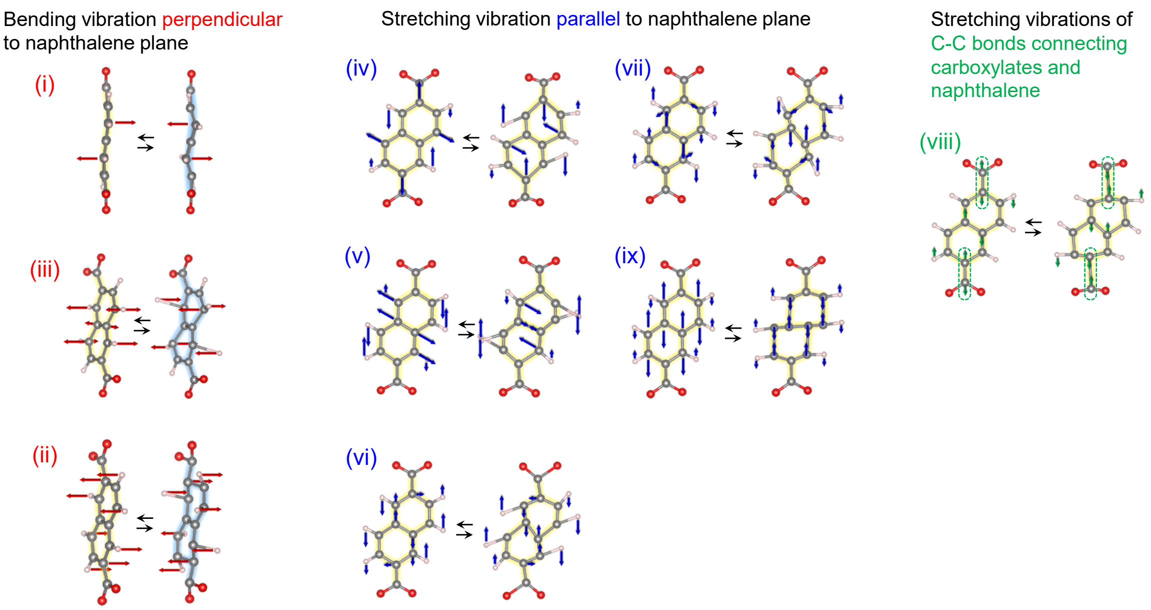

Supplementary Fig. 25. Representative vibration modes predicted by phonons using first principles calculations at the peaks shown in Supplementary Fig. 24.

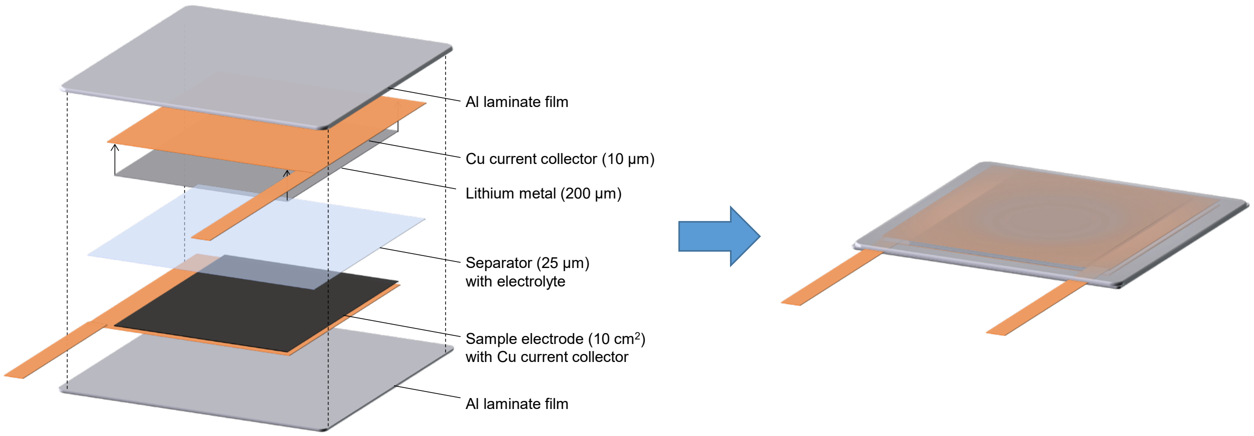

Supplementary Fig. 26. Schematic illustration of laminated-type cell before and after assembly, including information on each component.

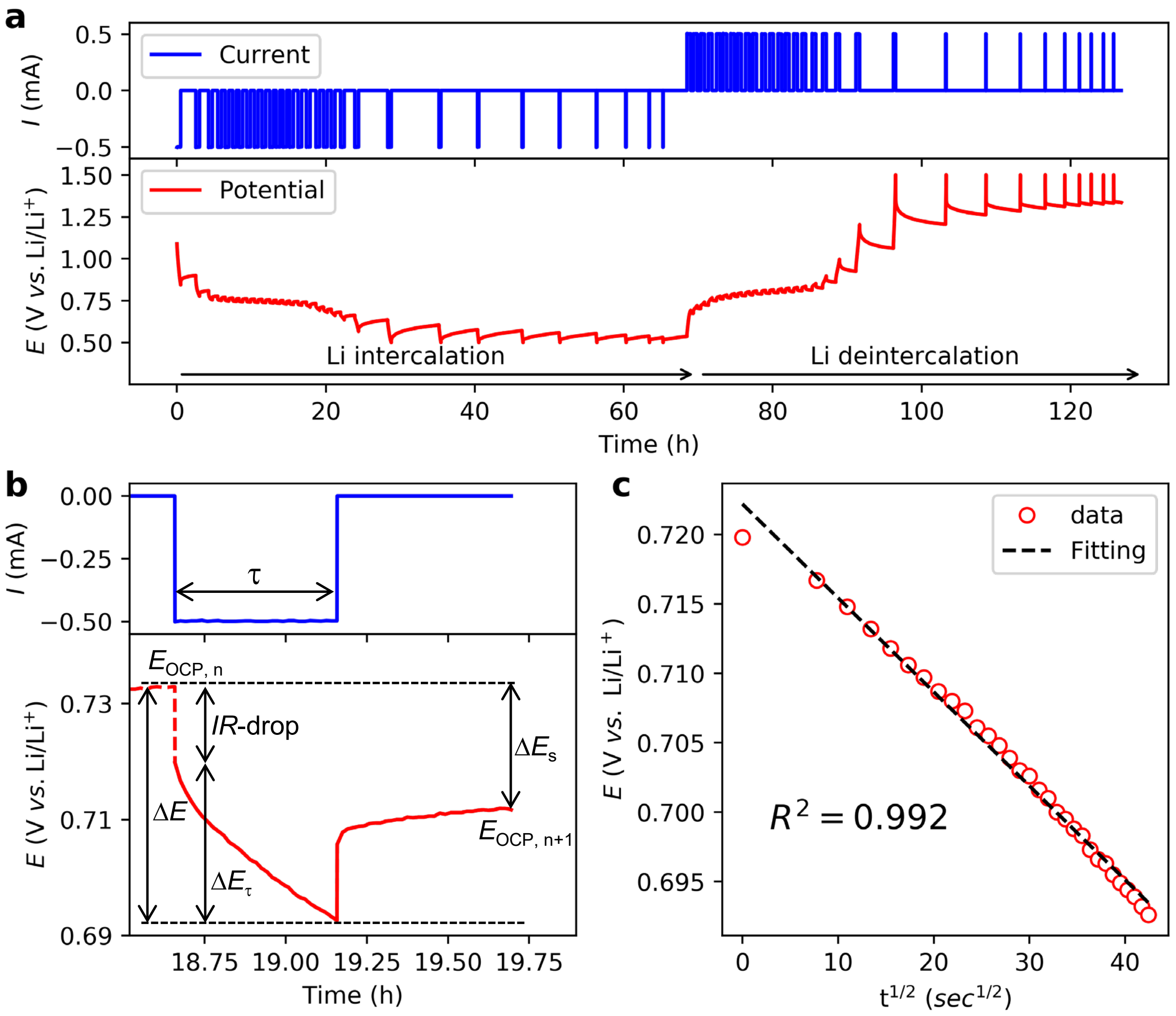

Supplementary Fig. 27. Schematic illustration of the galvanostatic intermittent titration technique (GITT). a Overall applied current and voltage output variation in the GITT with a steady-state laminate-type pouch Li||SD-NBP(762202) cell using the LiFSI-based electrolyte during Li intercalation and deintercalation at 20°C. **b** Applied current and voltage output variation for a single step. **c** Representation of the transient voltage as a function of the square root of time. Dotted lines are the results of linear approximation; R^2 is the coefficient of determination in the linear approximation.

Supplementary Table 1. Temperature and time for the heat treatment of each electrode active material at specific conditions.

Type	Active material	Heat treatment temperature (°C)	Heat treatment time (h)	Ref.
iMOF	Bulk-iMOFs	120	6	4
	SD-iMOFs	120	1	2
Organic	dihydroxy-benzoquinone	60	12	5
	sulfonamide	115	20	6
	triquinoxalinylene	140	3	7
	naphtalene diimide	150	19	8
	enolate	250	16	9
Negative	Natural or artificial graphite carbon	2700-3000	3	10
	MXene (precursor synthesis temp. of Ti_2AlC) [a]	1600	10	11
	MXene (precursor synthesis temp. of Ti_3AlC_2) [a]	1350	2	12
	$TiNb_2O_7$	1100	12	13
	Hard carbon	1000	6	14
	$Li_4Ti_5O_{12}$	800	12	15
	Nb_2O_5-rGO	500	6	16
	orthorhombic Nb_2O_5	600	2	17
Positive	$LiNi_{1/3}Mn_{1/3}Co_{1/3}O_2$	1000	12	18
	$LiNiO_2$	750	24	19
	$LiNi_{0.5}Mn_{1.5}O_4$	1000	12	20
	$LiMn_2O_4$	900	10	21
	$LiNi_{0.75}Co_{0.15}Al_{0.05}Mg_{0.05}O_2$	850	5	22
	$LiCoO_2$	850	24	23
	$LiFePO_4$	800	24	24
	$LiNi_{0.8}Co_{0.2}O_2$	800	20	25

[a] Heat-treatment temperatures during precursor synthesis.

Supplementary Table 2. Specific surface areas and pore data for pristine samples.

	SD-NBP(750718)		SD-NBP(762202)		SD-Naph
	Cy	BF	Cy	BF	
S_{BET} [a](m^2 g^{-1})	16.41	14.08	18.40	12.12	2.33
Avg. pore size$_{BJH}$ [b](nm)	13.87	13.61	11.77	15.20	24.49
V_{BJH} [b] (cm^3 g^{-1})	0.060	0.053	0.059	0.050	0.013

[a] Brunauer–Emmett–Teller surface area (S_{BET}) was calculated from the BET plot in the region of 0-0.005 p/p_0. Measurement error is 0.01 m^2 g^{-1}.

[b] Avg. pore size$_{BJH}$ and pore volume (V_{BJH}) were calculated by the Barrett–Joyner–Halenda (BJH) method.

Supplementary Table 3. Estimated results for unit cell parameters and volumes (V) of pristine samples.

	SD-Naph	SD-NBP(750025)	SD-NBP(750718)	SD-NBP(762202)
Cell setting	Monoclinic	Monoclinic	Monoclinic	Monoclinic
Space group	$P2_1/C$	$P2_1/C$	$P2_1/C$	$P2_1/C$
a (Å)	10.29	10.28	10.53	10.50
b (Å)	5.34	5.33	5.26	5.27
c (Å)	8.63	8.62	8.83	8.80
β (°)	98.73	98.72	94.63	94.83
V (Å^3)	468.7	467.2	487.9	485.2

Supplementary Table 4. List of electrode performances of reported MOFs.

No	Sample (Active material ratio, wt.%)	Electrolyte	Specific current ($mA\ g^{-1}$)	Reversible capacity ($mA\ h\ g^{-1}$)	Charging time (min.) [a]	Loading weight ($mg\ cm^{-2}$)	Testing temp. (°C)	Ref
	Negative							
1	SD-NBP (71.4)	LiFSI/ EC+DMC+EMC (3+4+3)	400	190	29	2.6	20	This work
2	Co-COP (70)	$LiPF_6$/ EC+DEC [c] (1+1)	500	920	110	1.5	–	26
3	Mn-BTC (70)	$LiPF_6$/ EC+DMC (1+1)	206	694	202	0.8	–	27
4	Ni-MOF (70)	–	250	654	157	1.0	–	28
5	CoBTC (70)	$LiPF_6$/ EC+DEC+DMC (1+1+1)	200	856	257	2.5	–	29
6	Co-TFBTC (70)	$LiPF_6$/ EC+ DEC+EMC (1+1+1)	200	939	282	1.0	r. t. [b]	30
7	Fe-BTC (70)	$LiPF_6$/ EC+DEC+EMC (1+1+1)	200	990	297	2.0	–	31
8	Mn-PBA (70)	$LiPF_6$/ EC+DMC+EMC (1+1+1)	100	503	302	0.7	–	32
9	GCP350 (–)	$LiPF_6$/ EC+DMC (1+1)	200	1188	356	1.5	–	33
10	FOR1 (70)	$LiPF_6$/ EC+DMC (1+1)	60	600	600	1.3	r. t. [b]	34
	Positive							
11	Cu-TCA (80)	$LiPF_6$/ EC+DMC (1+1)	50.1	102.2	122	0.5	–	35
12	$FeFe(CN)_6$ (70)	$LiPF_6$/ EC+DEC (1+1)	50	160	192	3.0	–	36
13	MIL-47 (65)	$LiPF_6$/ EC+DMC (1+1)	12	110	550	1.4	r. t. [b]	37
14	$Cu_3(HHTP)_2$ (60)	$Zn(CF_3SO_3)_2$/ acetonitrile	100	191	115	2.0	25	38

[a] C rate = Reversible capacity ($mA\ h\ g^{-1}$) / Specific current ($mA\ g^{-1}$)

[b] Room temperature. [c] DEC: Diethyl Carbonate

The counter electrodes are all Li metal, except for Zn metal in No. 14.

The reversible capacities are the initial values at the specific currents.

Supplementary Table 5. Estimated results for unit cell parameters and volumes (V) at electrodes before and after Li intercalation obtained from ex-situ synchrotron XRD patterns.

	SD-Naph			SD-NBP(762202)		
Cell setting	Before	After	Δ (%)	Before	After	Δ (%)
Space group	$P2_1/C$	$P2_1/C$		$P2_1/C$	$P2_1/C$	
a (Å)	10.25	9.66	-5.781	10.42	10.04	-3.666
b (Å)	5.33	5.99	12.394	5.24	5.70	8.779
c (Å)	8.62	8.37	-2.887	8.74	9.10	4.151
β (°)	98.76	105.32	6.642	95.97	107.88	12.410
V (Å^3)	465.1	466.7	0.356	474.1	495.2	4.435

Supplementary References

1. Hazama, H., Murai, D., Nagasako, N., Hasegawa, M. & Ogihara, N. Optimization of material composition of li-intercalated metal-organic framework electrodes using a combination of experiments and machine learning of X-ray diffraction patterns. *Adv. Mater. Technol.* **5,** 2000254 (2020).
2. Ogihara, N., Hasegawa, M., Kumagai, H. & Nozaki, H. Low-Resistance Mechanism of Nanoflake Crystalline Aromatic Dicarboxylates with Selective Defects for Safe and Fast Charging Negative Electrodes. *ACS Nano* **15,** 2719-2729 (2021).
3. Shiraishi, S., Kurihara, H., Shi, L., Nakayama, T. & Oya, A. Electric double-layer capacitance of meso/macroporous activated carbon fibers prepared by the blending method - I. Nickel-loaded activated carbon fibers in propylene carbonate solution containing $LiClO_4$ salt. *J. Electrochem. Soc.* **149,** A855-A861 (2002).
4. Ozawa, Y. et al. Intercalated metal-organic frameworks with high electronic conductivity as negative electrode materials for hybrid capacitors. *Commun. Chem.* **1,** 65 (2018).
5. Wu, X. et al. Unraveling the storage mechanism in organic carbonyl electrodes for sodium-ion batteries. *Sci. Adv.* **1,** e1500330 (2015).
6. Wang, J. et al. Conjugated sulfonamides as a class of organic lithium-ion positive electrodes. *Nat Mater* **20,** 665-673 (2021).
7. Peng, C. et al. Reversible multi-electron redox chemistry of π-conjugated N-containing heteroaromatic molecule-based organic cathodes. *Nat. Energy* **2,** 17074 (2017).
8. Lakraychi, A. E. et al. Carboxylic and sulfonic N-substituted naphthalene diimide salts as highly stable non-polymeric organic electrodes for lithium batteries. *Electrochem. Commun.* **76,** 47-50 (2017).
9. Gottis, S., Barres, A. L., Dolhem, F. & Poizot, P. Voltage gain in lithiated enolate-based organic cathode materials by isomeric effect. *ACS Appl. Mater. Interfaces* **6,** 10870-10876 (2014).
10. Dahn, J. R., Zheng, T., Liu, Y. H. & Xue, J. S. Mechanisms for Lithium Insertion in Carbonaceous Materials. *Science* **270,** 590-593 (1995).
11. Naguib, M. et al. Two-Dimensional Transition Metal Carbides. *ACS Nano* **6,** 1322-1331 (2012).
12. Ghidiu, M., Lukatskaya, M. R., Zhao, M. Q., Gogotsi, Y. & Barsoum, M. W. Conductive two-dimensional titanium carbide 'clay' with high volumetric capacitance. *Nature* **516,** 78-81 (2014).
13. Takami, N. et al. High-energy, fast-charging, long-life lithium-ion batteries using $TiNb_2O_7$ anodes for automotive applications. *J. Power Sources* **396,** 429-436 (2018).
14. Buiel, E., George, A. E. & Dahn, J. R. On the reduction of lithium insertion capacity in hard-carbon anode materials with increasing heat-treatment temperature. *J. Electrochem. Soc.* **145,** 2252-2257 (1998).
15. Ohzuku, T., Ueda, A. & Yamamota, N. Zero-strain insertion material of $Li[Li_{1/3}Ti_{5/3}]O_4$ for rechargeable

lithium cells. *J. Electrochem. Soc.* **142,** 1431-1435 (1995).

16. Lai, C. H. et al. Designing Pseudocapacitance for Nb_2O_5/Carbide-Derived Carbon Electrodes and Hybrid Devices. *Langmuir* **33,** 9407-9415 (2017).

17. Augustyn, V. et al. High-rate electrochemical energy storage through Li^+ intercalation pseudocapacitance. *Nat Mater* **12,** 518-522 (2013).

18. Yabuuchi, N., Makimura, Y. & Ohzuku, T. Solid-state chemistry and electrochemistry of $LiCo_{1/3}Ni_{1/3}Mn_{1/3}O_2$ for advanced lithium-ion batteries III. Rechargeable capacity and cycleability. *J. Electrochem. Soc.* **154,** A314-A321 (2007).

19. Ohzuku, T., Ueda, A. & Nagayama, M. Electrochemistry and structural chemistry of $LiNiO_2$ (R3m) for 4 volt secondary lithium cells. *J. Electrochem. Soc.* **140,** 1862-1870 (1993).

20. Ariyoshi, K., Iwakoshi, Y., Nakayama, N. & Ohzuku, T. Topotactic two-phase reactions of li [$Ni_{1/2}Mn_{3/2}$] O4 (*P*$4_3$32) in nonaqueous lithium cells. *J. Electrochem. Soc.* **151,** A296-A303 (2004).

21. Ben, L. et al. Unusual spinel-to-layered transformation in $LiMn_2O_4$ cathode explained by electrochemical and thermal stability investigation. *ACS Appl. Mater. Interfaces* **9,** 35463-35475 (2017).

22. Pouillerie, C., Croguennec, L., Biensan, P., Willmann, P. & Delmas, C. Synthesis and characterization of new $LiNi_{1-y}Mg_yO_2$ positive electrode materials for lithium ‐ ion batteries. *J. Electrochem. Soc.* **147,** 2061-2069 (2000).

23. Ohzuku, T. & Ueda, A. Solid-state redox reactions of $LiCoO_2$ (R3m) for 4 volt secondary lithium cells. *J. Electrochem. Soc.* **141,** 2972-2977 (1994).

24. Padhi, A. K., Nanjundaswamy, K. S. & Goodenough, J. B. Phospho-olivines as positive-electrode materials for rechargeable lithium batteries. *J. Electrochem. Soc.* **144,** 1188-1194 (1997).

25. Tang, H. W. et al. Synthesis and electrochemical properties of high-density $LiNi_{0.8}Co_{0.2}O_2$ for the lithium-ion-battery cathode. *Electrochem. Solid State Lett.* **11,** A34-A37 (2008).

26. Song, H., Shen, L., Wang, J. & Wang, C. Reversible lithiation–delithiation chemistry in cobalt based metal organic framework nanowire electrode engineering for advanced lithium-ion batteries. *J. Mater. Chem. A* **4,** 15411-15419 (2016).

27. Maiti, S., Pramanik, A., Manju, U. & Mahanty, S. Reversible Lithium Storage in Manganese 1,3,5-Benzenetricarboxylate Metal–Organic Framework with High Capacity and Rate Performance. *ACS Appl. Mater. Interfaces* **7,** 16357-16363 (2015).

28. Guo, L. et al. Construction of 1D conductive Ni-MOF nanorods with fast Li^+ kinetic diffusion and stable high-rate capacities as an anode for lithium ion batteries. *Nanoscale Advances* **1,** 4688-4691 (2019).

29. Li, C. et al. High Anodic Performance of Co 1,3,5-Benzenetricarboxylate Coordination Polymers for Li-Ion Battery. *ACS Appl. Mater. Interfaces* **8,** 15352-15360 (2016).

30. Lou, X. et al. Room-temperature synthesis of a cobalt 2,3,5,6-tetrafluoroterephthalic coordination polymer

with enhanced capacity and cycling stability for lithium batteries. *New J. Chem.* **41,** 1813-1819 (2017).

31. Hu, X. et al. Facile synthesis of the Basolite F300-like nanoscale Fe-BTC framework and its lithium storage properties. *RSC Advances* **6,** 114483-114490 (2016).
32. Xiong, P., Zeng, G., Zeng, L. & Wei, M. Prussian blue analogues $Mn[Fe(CN)_6]_{0.6667}\cdot nH_2O$ cubes as an anode material for lithium-ion batteries. *Dalton Transactions* **44,** 16746-16751 (2015).
33. Xiao, P. et al. Sub-5 nm Ultrasmall Metal–Organic Framework Nanocrystals for Highly Efficient Electrochemical Energy Storage. *ACS Nano* **12,** 3947-3953 (2018).
34. Saravanan, K., Nagarathinam, M., Balaya, P. & Vittal, J. J. Lithium storage in a metal organic framework with diamondoid topology – a case study on metal formates. *J. Mater. Chem.* **20,** 8329-8335 (2010).
35. Peng, Z., Yi, X., Liu, Z., Shang, J. & Wang, D. Triphenylamine-Based Metal–Organic Frameworks as Cathode Materials in Lithium-Ion Batteries with Coexistence of Redox Active Sites, High Working Voltage, and High Rate Stability. *ACS Appl. Mater. Interfaces* **8,** 14578-14585 (2016).
36. Zhang, K., Varma, R. S., Jang, H. W., Choi, J.-W. & Shokouhimehr, M. +Iron hexacyanocobaltate metal-organic framework: Highly reversible and stationary electrode material with rich borders for lithium-ion batteries. *J. Alloys Compd.* **791,** 911-917 (2019).
37. Kaveevivitchai, W. & Jacobson, A. J. Exploration of vanadium benzenedicarboxylate as a cathode for rechargeable lithium batteries. *J. Power Sources* **278,** 265-273 (2015).
38. Nam, K. W. et al. Conductive 2D metal-organic framework for high-performance cathodes in aqueous rechargeable zinc batteries. *Nat. Commun.* **10,** 4948 (2019).
